# Associations of maternal cyclin-dependent kinase 5 regulatory subunit-associated protein 1-like 1(*CDKAL1)* gene variants with adverse pregnancy outcome in Chinese women

**DOI:** 10.1186/s12884-025-07418-1

**Published:** 2025-03-25

**Authors:** Shuoying Yue, Meng Su, Zihao Zhang, Jing Li, Junhong Leng, Weiqin Li, Jin Liu, Tao Zhang, Yijuan Qiao, Zhijie Yu, Gang Hu, Jun Ma, Xilin Yang, Hui Wang

**Affiliations:** 1 Department of Epidemiology and Biostatistics, School of Public Health, Tianjin Medical University, Tianjin, China; 2 Tianjin Key Laboratory of Environment, Nutrition and Public Health, Tianjin, China; 3 Tianjin Center for International Collaborative Research on Environment, Nutrition and Public Health, Tianjin, China; 4 Project Office, Tianjin Women and Children’s Health Center, Tianjin, China; 5 Population Cancer Research Program, Department of Pediatrics, Dalhousie University, Halifax, NS Canada; 6 Chronic Disease Epidemiology Laboratory, Pennington Biomedical Research Center, Baton Rouge, LA USA

**Keywords:** *CDKAL1* gene, Adverse pregnancy outcome, Gestational diabetes mellitus, Low birth weight, Macrosomia

## Abstract

**Objective:**

To test associations of cyclin-dependent kinase 5 regulatory subunit-associated protein 1-like 1 (*CDKAL1*) gene variants with the risk of adverse pregnancy outcome in Chinese women and whether the association was mediated by occurrence of gestational diabetes mellitus.

**Methods:**

We organized a 1:1 age-matched study nested within a prospective cohort of pregnant women (207 pairs) established in urban Tianjin. Adverse pregnancy outcome was defined as a composite outcome of preterm birth, low birth weight or macrosomia. Logistic regression analyses were used to estimate associations of *CDKAL1* gene variants with adverse pregnancy outcome and its components. The *CDKAL1* genetic marker was defined as encompassing any of the identified susceptibility variants for adverse pregnancy outcome.

**Results:**

The *CDKAL1* genetic marker was associated with the risk of adverse pregnancy outcome (OR: 2.51, 95%CI: 1.47, 4.28), low birth weight (OR: 19.80, 95%CI: 2.15, 182) and macrosomia (OR: 2.40, 95%CI: 1.17, 4.93), but not with preterm birth (*P* = 0.105) after adjusting for traditional risk factors. Further adjusting for gestational diabetes mellitus, the *CDKAL1* genetic marker remained significantly associated with adverse pregnancy outcome, and the OR (95%CI) was 2.52 (1.48, 4.30).

**Conclusion:**

The maternal *CDKAL1* gene variants were associated with increased risk of adverse pregnancy outcome, low birth weight and macrosomia, independent of gestational diabetes mellitus. *CDKAL1* gene might be a useful marker for identification of individuals at a particularly high risk of adverse pregnancy outcome in early pregnancy.

**Supplementary Information:**

The online version contains supplementary material available at 10.1186/s12884-025-07418-1.

## Introduction

The cyclin-dependent kinase 5 regulatory subunit-associated protein 1-like 1 (*CDKAL1)* gene was associated with reduced pancreatic β-cell function, which affected insulin secretion in response to changes in plasma glucose levels [1]. It was confirmed in different populations, such as Koreans and Russians, that specific genetic variants of *CDKAL1* increased susceptibility to type 2 diabetes (T2D) and gestational diabetes mellitus (GDM) [2, 3]. Our study further verified that the *CDKAL1* rs7747752 increased the risk of GDM in Chinese women [4]. It was well-known that GDM increased the risk of adverse pregnancy outcomes, including shoulder dystocia, cesarean section, macrosomia, hypocalcemia, preterm birth, low birth weight, and others [5–8]. Additionally, GDM elevated the risk of developing diabetes in the mother later in life and increased the risk of obesity in the offspring [9–11]. Several studies explored the association between fetal *CDKAL1* genotype (rather than maternal genotype) and their birth weight, but the conclusions were inconsistent [12, 13]. However, no studies have focused on whether maternal *CDKAL1* genetic variants increased the risk of adverse pregnancy outcomes. Therefore, it is essential to explore whether the maternal *CDKAL1* gene variants increased the risk of adverse pregnancy outcomes and whether this process was mediated through GDM.

Our study aimed to test the association of maternal *CDKAL1* gene variants with the risk of adverse pregnancy outcome (APO), including any of preterm birth, low birth weight, or macrosomia, and whether it was mediated by GDM, based on a previous nested case–control study in a prospective cohort of pregnant women with GDM in Tianjin, China [14].

## Method

### Research design and participants

The design and population of the study have been described previously [14]. We established a prospective cohort of 22,302 pregnant women from 6 urban districts of Tianjin, China from October 2010 to August 2012. This research protocol was approved by the Clinical Research Ethics Committee of Tianjin Women’s and Children’s Health Center (TWCHC). Written informed consent was obtained from the participants before data collection. This study was performed in line with the principles of the Declaration of Helsinki.

A tiered screening strategy was employed to identify cases of GDM. Initially, at primary healthcare institutions, all participants were invited to complete a 1-h 50-g oral glucose challenge test (GCT) at 24–28 weeks of gestation. Participants exhibiting GCT results ≥ 7.8 mmol/L were subsequently referred to a dedicated GDM clinic within TWCHC for a more comprehensive assessment. At this stage, they underwent a 2-h 75-g oral glucose tolerance test (OGTT) after more than 8 h of fasting. The diagnostic criteria for GDM adhered to the International Association of Diabetes and Pregnancy Study Groups (IADPSG), which encompassed a fasting plasma glucose (PG) ≥ 5.1 mmol/L, a 1-h PG ≥ 10.0 mmol/L, or a 2-h PG ≥ 8.5 mmol/L [15].

**Fig. 1:**
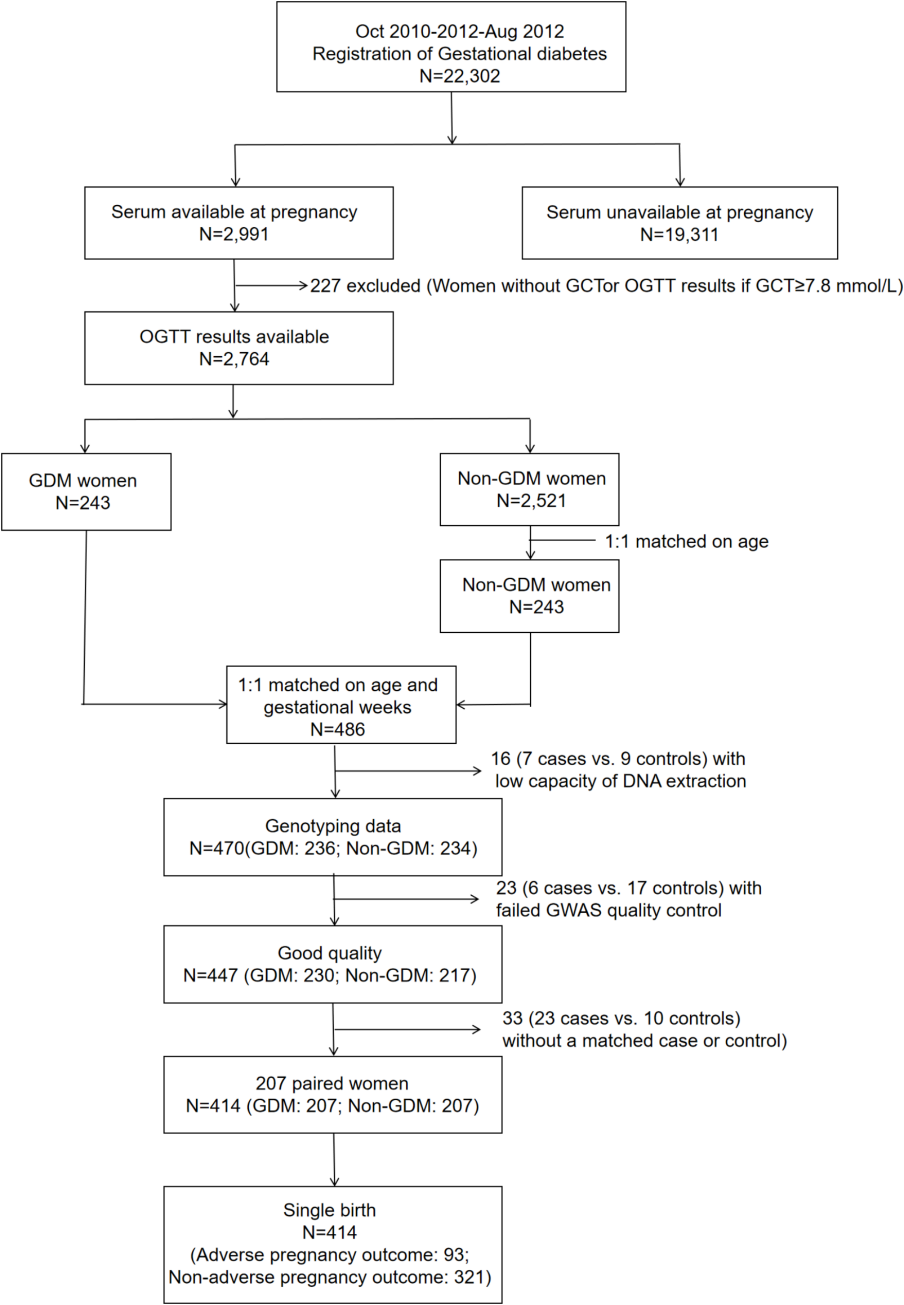
Title: Patient flowchart Legends: Adverse pregnancy outcome included any of preterm birth, low birth weight, or macrosomia Abbreviations: GCT, glucose challenge test; OGTT, oral-glucose-tolerance test; GDM, gestational diabetes mellitus; DNA, Deoxyribonucleic Acid; GWAS, genome-wide association study

In this cohort, 2,991 women out of 22,302 pregnant donated their blood samples during early pregnancy at the early stage of the study. Among the remaining 2,764 participants with OGTT results available, 243 women with GDM were used as the study cases and 243 healthy women with matched maternal ages (± 1 y) were selected as the controls. Among the 486 women, 16 women with low capacity of DNA extraction, 23 women who lacked high-quality DNA data and 33 women who did not have an age-matched GDM counterpart or control were excluded. The remaining 207 pairs of GDM patients and controls (*n* = 414) were eligible for this study (Fig. 1).

### Data collection procedure

We collected pregnancy information on height, weight, maternal age, systolic/diastolic blood pressure (BP), parity, ethnicity, family history of diabetes in first-degree relatives, education level, and gestational age at registration. Besides we collected delivery information including infant gender, birth weight of neonates, body height of neonates, and delivery week. Pre-pregnancy body mass index (BMI) (kg/m^2^) was estimated as pre-pregnancy weight (kg) divided by height squared (m^2^). Hypertension was defined as systolic BP ≥ 140 mmHg or diastolic BP ≥ 90 mmHg [16].

### Ascertainment of APO

APO was defined as any of preterm birth, low birth weight, or macrosomia in our study. Preterm birth was defined as delivery week < 37 weeks [17]. Low birth weight was identified as birth weight of neonates < 2,500 g and macrosomia referred to ≥ 4,000 g [18].

### Genotyping

Blood samples were collected in the fasting state at registration and stored at − 80℃ until use. DNA samples were genotyped by the Illumina Infinium^®^ Global Screening Array. The genotype data were imputed using minimac 3 with the 1000 Genomes Project phase 3V5 as a reference panel. Based on the single nucleotide polymorphism (SNP) database, the following quality control steps were implemented: (1) Samples with a call rate < 97% were filtered out; (2) SNPs with > 20% missing data and individuals with > 2% missing genotype data were removed; (3) Subjects with discrepant gender information were excluded; (4) Individuals with a minor allele frequency < 1% were filtered out; (5) SNPs with a Hardy-Weinberg equilibrium P-value < 1 × 10^− 4^ were filtered out; (6) Individuals deviating from the mean heterozygosity rate by ± 3 standard deviations (SD) were excluded; (7) Relatedness and ancestry outliers (pi-hat > 0.2, concordance > 0.98) were identified and filtered out [19]. Genotyping data from specific candidate SNP (*CDKAL1* gene variants) were extracted from the genome-wide genotyping. The overall genotype call rate was 99.4%.

### Statistical analysis

Continuous data were presented as mean ± SD or median [interquartile range, (IQR)] and compared by t-test or Wilcoxon rank sum test, while categorical variables were presented by number (percentage) and compared by chi-square tests or Fisher’s exact test. Firstly, logistic regression analyses were used to estimate the odds ratios (ORs) and corresponding 95% confidence intervals (95%CIs) of *CDKAL1* gene variants on the risk of APO and its components, including preterm birth, low birth weight, and macrosomia. The *CDKAL1* genetic marker was defined as encompassing any one of identified susceptibility variants related to APO, and accordingly, ORs and their corresponding 95%CIs for the risk of APO and its components were calculated using logistic regression analyses.

To control for potential confounding factors associated with APO, traditional risk factors, including age, pre-pregnancy BMI, family history of diabetes in first degree relatives, parity, education, Han ethnicity, hypertension, infant gender, and body height of neonates, were adjusted in the adjusted model 1. Further adjusted for GDM in the adjusted model 2 to explore the mediation effects of GDM on the association of *CDKAL1* genetic marker and APO.

Analysis was performed using R (R Core Team, 2024. R Foundation for Statistical Computing, Vienna, Austria.). A P value < 0.05 was considered to be statistically significant.

## Results

### Characteristics of the participants with APO

For the 414 pregnant women included in this study, the median age was 29 years and the median gestational age was 10 weeks at registration. There were 93 women who experienced an APO among the included participants. Compared to women without APO, women with APO had a higher pre-pregnancy BMI and a lower value of delivery week. Women with APO were more likely to have genotypes AG/AA for *CDKAL1* rs141859146 and CT/CC for rs7762612. And the remaining 21 maternal *CDKAL1* gene variants had no statistical differences in the women with and without APO group (Table 1 and Appendix Table 1).

**Table 1:** Clinical characteristics of women with APO

Characteristic	Non-APO women	APO women	*P*
	(*n* = 321)	(*n* = 93)	
**Variables during pregnancy**			
Age, years	29 [27,31]	29 [27,31]	0.827
Pre-pregnancy BMI, kg/m^2^	22.1 [20.0,24.9]	23.9 [21.6,26.2]	0.001
Systolic BP, mmHg	105 [100,110]	110 [100,120]	0.125
Diastolic BP, mmHg	70 [60,70]	70 [60,75]	0.119
Hypertension	4 (1.2)	4 (4.3)	0.145
Han ethnicity	310 (96.6)	92 (98.9)	0.401
Education > 12 years	180 (56.1)	47 (50.5)	0.409
Parity ≥ 1	15 (4.7)	8 (8.6)	0.230
Family history of diabetes in first degree relatives	29 (9.0)	10 (10.8)	0.766
GDM	155 (48.3)	52 (55.9)	0.239
Gestational age at registration, weeks	10.0 [9.0,11.0]	10.0 [9.0,12.0]	0.771
***CDKAL1*** **gene variants**			
rs141859146(A/G)			0.049
GG	315 (98.1)	87 (93.5)	
AG/AA	6 (1.9)	6 (6.5)	
rs7762612(C/T)			0.015
TT	272 (84.7)	68 (73.1)	
CT/CC	49 (15.3)	25 (26.9)	
rs4710944(T/C)			0.076
CC	312 (97.2)	86 (92.5)	
TC/TT	9 (2.8)	7 (7.5)	
**Variables during delivery**			
Infant female gender	140 (43.6)	33 (35.5)	0.200
Birth weight of neonates, g	3400 [3100,3650]	4050 [2850,4300]	< 0.001
Body height of neonates, cm	50 [50,51]	51 [49,52]	0.011
Delivery week, weeks	39.0 [38.0,40.0]	38.0 [36.0,40.0]	< 0.001

Abbreviations: APO, adverse pregnancy outcome; BMI, body mass index; BP, blood pressure; GDM, gestational diabetes mellitus; *CDKAL1*, cyclin-dependent kinase 5 regulatory subunit associated protein 1-like 1

Data was reported n (%) or medians (IQRs)

P for continuous variables derived from Wilcoxon rank sum test and for categorical variables derived from Chi-square test or Fisher’s exact test

### Associations of maternal *CDKAL1* gene variants on the risk of APO

In the dominant model, maternal *CDKAL1* rs141859146 (AG/AA vs. GG), rs7762612 (CT/CC vs. TT) and rs4710944 (TC/TT vs. CC) were associated with the risk of APO in the univariate analyses, with the ORs (95%CIs) were 3.62 (1.14, 11.5), 2.04 (1.18, 3.54), and 2.82 (1.02, 7.80) respectively (Table 2). Other remaining 20 maternal *CDKAL1* gene variants were not associated with the risk of APO (Appendix Table 2).

**Table 2:** Unadjusted odds ratios of maternal *CDKAL1* gene variants on APO

*CDKAL1*	OR (95% CI)	*P*
*CDKAL1* gene variants		
rs141859146(A/G)		
AG/AA vs. GG	3.62 (1.14,11.5)	0.029
rs7762612(C/T)		
CT/CC vs. TT	2.04 (1.18,3.54)	0.011
rs4710944(T/C)		
TC/TT vs. CC	2.82 (1.02,7.80)	0.045
*CDKAL1* genetic marker		
any of above variants	2.34 (1.41,3.90)	0.001

Abbreviations: APO, adverse pregnancy outcome; *CDKAL1*, cyclin-dependent kinase 5 regulatory subunit associated protein 1-like 1; OR, odds ratio; CI, confidence interval

### Maternal *CDKAL1* genetic marker on APO and its components

*CDKAL1* genetic marker was defined as encompassing any of the susceptibility variants rs141859146, rs7762612 or rs4710944 for APO. Compared to women without *CDKAL1* genetic marker, women with *CDKAL1* genetic marker were more likely to have APO and had higher birth weight and height of neonates. There were no statistical differences in other characteristics, such as maternal age and pre-pregnancy BMI, etc., between the groups with and without the *CDKAL1* genetic marker (Appendix Table 3).

**Table 3:** Odds ratio of maternal *CDKAL1* genetic marker on APO and its components

Outcomes	Adjusted model 1		Adjusted model 2	
	OR (95% CI)	*P*	OR (95% CI)	*P*
**APO**	2.51 (1.47,4.28)	< 0.001	2.52 (1.48,4.30)	< 0.001
**Birth weight of neonates**				
Normal birth weight	-	-	-	-
Low birth weight	19.80 (2.15,182)	0.008	20.62 (2.07,205)	0.010
Macrosomia	2.40 (1.17,4.93)	0.017	2.39 (1.16,4.91)	0.018
**Preterm birth**	2.28 (0.84,6.19)	0.105	2.36 (0.86,6.43)	0.094

Abbreviations: APO, adverse pregnancy outcome; *CDKAL1*, cyclin-dependent kinase 5 regulatory subunit associated protein 1-like 1; OR, odds ratio; CI, confidence interval

Adjusted model 1, adjusted for age, pre-pregnancy body mass index, family history of diabetes in first degree relatives, parity, education, Han ethnicity, hypertension, infant gender, and body height of neonates

Adjusted model 2, further adjusted for gestational diabetes mellitus, in addition to the variables in the adjusted model 1

Maternal *CDKAL1* genetic marker was significantly associated with the risk of APO, with the OR (95%CI) was 2.34 (1.41, 3.90) in the unadjusted model (Table 2).

*CDKAL1* genetic marker was also associated with a significantly elevated risk of APO (OR: 2.51, 95%CI: 1.47, 4.28) after adjustment for traditional risk factors in the adjusted model 1. After further adjusting for GDM, the *CDKAL1* genetic marker exhibited a comparable risk for APO, with the OR (95%CI) was 2.52 (1.48, 4.30) (Table 3).

The *CDKAL1* genetic marker was associated with the risk of low birth weight (OR: 19.80, 95%CI: 2.15, 182) and macrosomia (OR: 2.40, 95%CI: 1.17, 4.93) but had no significant association with preterm birth (*P* = 0.105), after adjusting for traditional risk factors. After adjusting for GDM alongside the traditional risk factors, the *CDKAL1* genetic marker remained significantly associated with low birth weight and macrosomia, with the ORs showing no significant alterations. The adjusted ORs (95%CIs) were 20.62 (2.07, 205) for low birth weight, and 2.39 (1.16, 4.91) for macrosomia, respectively (Table 3).

## Discussion

Our study revealed that the maternal *CDKAL1* gene variants was associated with the risk of APO and its components, including low birth weight and macrosomia, but not with preterm birth. Notably, this association was independent of GDM.

As a key risk factor gene for GDM, the potential associations of *CDKAL1* gene with both the short-term and long-term health outcomes of GDM should be a top priority in scientific research. However, this issue had not been explored in studies thus far. Currently, there were some studies focusing on the associations of the fetal *CDKAL1* gene with their birth weight, but the conclusions were inconsistent. An ongoing genome-wide association study based on 5,465 Caucasian children showed that fetal *CDKAL1* rs7756992 was strongly associated with low birth weight [20], while a study based on the Mexican population indicated that rs7754840 in *CDKAL1* was not associated with birth weight [13]. After adjusting for confounding factors such as maternal age and BMI, we found that the maternal *CDKAL1* gene variants were significantly associated with APO, particularly the risks of low birth weight and macrosomia. Even after further adjusting for GDM, this association remained independent, indicating that the association between the *CDKAL1* gene and birth weight was independent of GDM. The biological mechanism by which the maternal *CDKAL1* gene influenced APO was still unclear, possibly involving its impact on insulin secretion and metabolic pathways, thereby altering blood glucose levels. Maternal blood glucose levels might potentially affect fetal growth and delivery outcomes [21]. Further research was needed to elucidate this mechanism. This new finding deepened our understanding of the genetic factors influencing pregnancy outcomes and emphasized the importance of considering genetic variants in research on maternal health.

The identification of the maternal *CDKAL1* gene variants as a risk factor for APO, independent of GDM, had important public health implications. Low birth weight was associated with postnatal metabolic disorders (such as obesity and insulin resistance), cardiovascular disease in adults, and diabetes [22, 23]. Similarly, preterm birth was considered a risk factor for diabetes [24] and macrosomia was regarded as a factor increased the risk of obesity later in life [25]. The detection of *CDKAL1* gene variants in pregnant women facilitated identifying pregnant women those at a high risk of APO, which could help prevent and manage APOs, through monitoring intrauterine growth and development of newborns and providing targeted prenatal care.

The study had several strengths and limitations. The strength was that it was a real-world cohort of pregnant women, including genetic variation and offspring information for pregnant women. A limitation of this study was that it was based on a cohort of pregnant women in Tianjin, and the research findings needed to be validated in other populations. Additionally, the IADPSG recommended using a one-step OGTT method to identify GDM, whereas we employed a two-step procedure for screening GDM in the current study, which might result in some pregnant women being missed. During our analysis, we accounted for internal environmental factors, including maternal age and pre-pregnancy BMI. However, owing to the lack of external environmental data in our database, we did not consider factors such as particulate matter 2.5, carbon dioxide, and other environmental variables.

In conclusion, *CDKAL1* gene variants were associated with the risk of APO, low birth weight and macrosomia, independent of GDM. *CDKAL1* gene variants might be useful markers for APO, low birthweight and macrosomia. Further research was warranted to explore the cause-effect association and underlying mechanisms between *CDKAL1* gene variants and fetal growth and development, which might lead to the discovery of new therapeutic strategies, such as the influence of phytochemicals on *CDKAL1* gene expression [26], thereby reducing the risk of APO in Chinese pregnant women.

## Electronic supplementary material

Below is the link to the electronic supplementary material.

Supplementary Material 1

## Data Availability

The datasets used and/or analyzed during the current study are available from the corresponding author on reasonable request.

## Abbreviations

*CDKAL1*: cyclin-dependent kinase 5 regulatory subunit-associated protein 1-like 1
T2D: type 2 diabetes
GDM: gestational diabetes mellitus
APO: adverse pregnancy outcome
TWCHC: Clinical Research Ethics Committee of Tianjin Women's and Children's Health Center
GCT: glucose challenge test
OGTT: oral glucose tolerance test
IADPSG: International Association of Diabetes and Pregnancy Study Groups
BP: blood pressure
BMI: body mass index
SNP: single nucleotide polymorphism
SD: standard deviation
IQR: interquartile range
OR: odds ratio
CI: confidence interval

## References

[1] Pascoe L, Tura A, Patel SK, Ibrahim IM, Ferrannini E, Zeggini E, et al. Common variants of the novel type 2 diabetes genes CDKAL1 and HHEX/IDE are associated with decreased pancreatic β-Cell function. Diabetes. 2007;56:3101–4. 17804762 10.2337/db07-0634

[2] Association between polymorphisms in SLC30A8, HHEX, CDKN2A/B, IGF2BP2., FTO, WFS1, CDKAL1, KCNQ1 and type 2 diabetes in the Korean population| Journal of Human Genetics. https://www.nature.com/articles/jhg2008127. Accessed 30 Nov 2024. 10.1007/s10038-008-0341-8 18991055

[3] Chistiakov DA, Potapov VA, Smetanina SA, Bel’chikova LN, Suplotova LA, Nosikov VV. The carriage of risk variants of CDKAL1 impairs beta-cell function in both diabetic and non-diabetic patients and reduces response to non-sulfonylurea and sulfonylurea agonists of the pancreatic KATP channel. Acta Diabetol. 2011;48:227–35. 21611789 10.1007/s00592-011-0299-4

[4] Wang H, Yang W, Liu J, Leng J, Li W, Yu Z, et al. Serum concentrations of SFAs and CDKAL1 single-nucleotide polymorphism rs7747752 are related to an increased risk of gestational diabetes mellitus. Am J Clin Nutr. 2021;114:1698–707. 34192303 10.1093/ajcn/nqab225

[5] Pan Y, Huang X, Jiang X. Risk factors and prediction model for low-birth-weight infants born to women with gestational diabetes mellitus. Front Public Health. 2024;12:1432033. 10.3389/fpubh.2024.1432033 PMC11514069 39469216

[6] Ye W, Luo C, Huang J, Li C, Liu Z, Liu F. Gestational diabetes mellitus and adverse pregnancy outcomes: systematic review and meta-analysis. BMJ. 2022;377:e067946. 35613728 10.1136/bmj-2021-067946 PMC9131781

[7] La Verde M, De Franciscis P, Torre C, Celardo A, Grassini G, Papa R, et al. Accuracy of fetal biacromial diameter and derived ultrasonographic parameters to predict shoulder dystocia: A prospective observational study. Int J Environ Res Public Health. 2022;19:5747. 35565142 10.3390/ijerph19095747 PMC9101462

[8] Deepa R, Van Schayck OCP, Babu GR. Low levels of vitamin D during pregnancy associated with gestational diabetes mellitus and low birth weight: results from the MAASTHI birth cohort. Front Nutr. 2024;11:1352617. 38887504 10.3389/fnut.2024.1352617 PMC11180835

[9] Catalano P, deMouzon SH. Maternal obesity and metabolic risk to the offspring: why lifestyle interventions May have not achieved the desired outcomes. Int J Obes. 2015;39:642–9. 10.1038/ijo.2015.15 PMC4700513 25777180

[10] La Verde M, Torella M, Riemma G, Narciso G, Iavarone I, Gliubizzi L, et al. Incidence of gestational diabetes mellitus before and after the Covid-19 lockdown: A retrospective cohort study. J Obstet Gynecol Res. 2022;48:1126–31. 35199420 10.1111/jog.15205 PMC9115303

[11] Sheiner E. Gestational diabetes mellitus: Long-Term consequences for the mother and child grand challenge: how to move on towards secondary prevention?? Front Clin Diabetes Healthc. 2020;1:546256. 36993989 10.3389/fcdhc.2020.546256 PMC10041873

[12] Sun X-F, Xiao X-H, Zhang Z-X, Liu Y, Xu T, Zhu X-L, et al. Positive association between type 2 diabetes risk alleles near CDKAL1 and reduced birthweight in Chinese Han individuals. Chin Med J. 2015;128:1873–8. 26168825 10.4103/0366-6999.160489 PMC4717941

[13] Aguilera-Venegas I-G, Mora-Peña J-S, Velazquez-Villafaña M, Gonzalez-Dominguez M-I, Barbosa-Sabanero G, Gomez-Zapata H-M, et al. Association of diabetes-related variants in ADCY5 and CDKAL1 with neonatal insulin, C-peptide, and birth weight. Endocrine. 2021;74:318–31. 34169461 10.1007/s12020-021-02799-7

[14] Leng J, Shao P, Zhang C, Tian H, Zhang F, Zhang S, et al. Prevalence of gestational diabetes mellitus and its risk factors in Chinese pregnant women: A prospective Population-Based study in Tianjin, China. PLoS ONE. 2015;10:e0121029. 25799433 10.1371/journal.pone.0121029 PMC4370728

[15] International Association of Diabetes and Pregnancy Study Groups Consensus Panel. International association of diabetes and pregnancy study groups recommendations on the diagnosis and classification of hyperglycemia in pregnancy. Diabetes Care. 2010;33:676–82. 20190296 10.2337/dc09-1848 PMC2827530

[16] Beaney T, Wang W, Schlaich MP, Schutte AE, Stergiou GS, Alcocer L, et al. Global blood pressure screening during the COVID-19 pandemic: results from the May measurement month 2021 campaign. J Hypertens. 2023;41:1446. 37337866 10.1097/HJH.0000000000003488 PMC10399936

[17] Hughes ZH, Hughes LM, Khan SS. Genetic contributions to risk of adverse pregnancy outcomes. Curr Cardiovasc Risk Rep. 2023;17:185–93. 38186860 10.1007/s12170-023-00729-y PMC10768680

[18] Chen X, Wang Q, Zang H, Cong X, Shen Q, Chen L. First trimester sCD40L levels associated with adverse neonatal outcomes in euthyroid pregnant women with positive TPOAb. Front Endocrinol (Lausanne). 2023;14:1097991. 10.3389/fendo.2023.1097991 PMC10243599 37288293

[19] Anderson CA, Pettersson FH, Clarke GM, Cardon LR, Morris AP, Zondervan KT. Data quality control in genetic case-control association studies. Nat Protoc. 2010;5:1564–73. 21085122 10.1038/nprot.2010.116 PMC3025522

[20] Zhao J, Li M, Bradfield JP, Wang K, Zhang H, Sleiman P, et al. Examination of type 2 diabetes loci implicates CDKAL1 as a birth weight gene. Diabetes. 2009;58:2414–8. 19592620 10.2337/db09-0506 PMC2750235

[21] Guo F, Long W, Zhou W, Zhang B, Liu J, Yu B. FTO, GCKR, CDKAL1 and CDKN2A/B gene polymorphisms and the risk of gestational diabetes mellitus: a meta-analysis. Arch Gynecol Obstet. 2018;298:705–15. 30074065 10.1007/s00404-018-4857-7

[22] Vejrazkova D, Lukasova P, Vankova M, Bradnova O, Vacinova G, Vcelak J, et al. Gestational Diabetes - Metabolic risks of adult women with respect to birth weight. Physiol Res. 2015;64:S135–45. 26680474 10.33549/physiolres.933089

[23] Sun Y, Wang B, Yu Y, Wang Y, Tan X, Zhang J, et al. Birth weight, ideal cardiovascular health metrics in adulthood, and incident cardiovascular disease. Chin Med J. 2024;137:1160. 38479998 10.1097/CM9.0000000000003043 PMC11101240

[24] Li S, Zhang M, Tian H, Liu Z, Yin X, Xi B. Preterm birth and risk of type 1 and type 2 diabetes: systematic review and meta-analysis. Obes Rev. 2014;15:804–11. 25073871 10.1111/obr.12214

[25] Gillman MW, Rifas-Shiman S, Berkey CS, Field AE, Colditz GA. Maternal gestational diabetes, birth weight, and adolescent obesity. Pediatrics. 2003;111:e221–6. 12612275 10.1542/peds.111.3.e221

[26] Ghosh C, Das N, Saha S, Kundu T, Sircar D, Roy P. Involvement of Cdkal1 in the etiology of type 2 diabetes mellitus and microvascular diabetic complications: a review. J Diabetes Metab Disord. 2022;21:991–1001. 35673487 10.1007/s40200-021-00953-6 PMC9167393

