# Supplementary material for: Associations of maternal cyclin-dependent kinase 5 regulatory subunit-associated protein 1-like 1(CDKAL1) gene variants with adverse pregnancy outcome in Chinese women
Source: BMC Pregnancy Childbirth. 2025 Mar 25;25:347. doi: 10.1186/s12884-025-07418-1 (PMC11934754; doi:10.1186/s12884-025-07418-1)
Supplement: Supplementary file 1 — Supplementary Material 1 [file 12884_2025_7418_MOESM1_ESM.docx]

Appendix Table 1. Characteristics of *CDKAL1* gene variants in APO group.

| Characteristic | Non-APO women | APO women | *P* |
| --- | --- | --- | --- |
|  | (n=321) | (n=93) |  |
| rs9368268(T/C) |  |  | 0.448 |
| CC | 226 (70.4) | 61 (65.6) |  |
| TC/TT | 95 (29.6) | 32 (34.4) |  |
| rs9350258(G/A) |  |  | 0.838 |
| AA | 85 (26.5) | 23 (24.7) |  |
| GA/GG | 236 (73.5) | 70 (75.3) |  |
| rs147434309(G/A) |  |  | 0.541 |
| AA | 269 (83.8) | 81 (87.1) |  |
| GA/GG | 52 (16.2) | 12 (12.9) |  |
| rs117779648(G/T) |  |  | 0.626 |
| TT | 293 (91.3) | 87 (93.5) |  |
| GT/GG | 28 (8.7) | 6 (6.5) |  |
| rs7747752(C/G) |  |  | 0.351 |
| GG | 79 (24.6) | 28 (30.1) |  |
| CG/CC | 242 (75.4) | 65 (69.9) |  |
| rs9358361(C/A) |  |  | 0.537 |
| AA | 197 (61.4) | 61 (65.6) |  |
| CA/CC | 124 (38.6) | 32 (34.4) |  |
| rs4585542(T/C) |  |  | 1.000 |
| CC | 116 (36.1) | 33 (35.5) |  |
| TC/TT | 205 (63.9) | 60 (64.5) |  |
| rs75239109(G/A) |  |  | 0.846 |
| AA | 314 (97.8) | 90 (96.8) |  |
| GA/GG | 7 (2.2) | 3 (3.2) |  |
| rs4712573(C/A) |  |  | 0.741 |
| AA | 116 (36.1) | 36 (38.7) |  |
| CA/CC | 205 (63.9) | 57 (61.3) |  |
| rs148988294(T/C) |  |  | 0.896 |
| CC | 307 (95.6) | 88 (94.6) |  |
| TC/TT | 14 (4.4) | 5 (5.4) |  |
| rs9465946(G/A) |  |  | 0.656 |
| AA | 239 (74.5) | 72 (77.4) |  |
| GA/GG | 82 (25.5) | 21 (22.6) |  |
| rs10946425(T/C) |  |  | 0.983 |
| CC | 189 (58.9) | 54 (58.1) |  |
| TC/TT | 132 (41.1) | 39 (41.9) |  |
| rs7768086(A/C) |  |  | 0.912 |
| CC | 189 (58.9) | 56 (60.2) |  |
| AC/AA | 132 (41.1) | 37 (39.8) |  |
| rs4712585(G/A) |  |  | 0.727 |
| AA | 286 (89.1) | 81 (87.1) |  |
| GA/GG | 35 (10.9) | 12 (12.9) |  |
| rs6937439(G/A) |  |  | 0.580 |
| AA | 112 (34.9) | 36 (38.7) |  |
| GA/GG | 209 (65.1) | 57 (61.3) |  |
| rs72838040(G/A) |  |  | 1.000 |
| AA | 295 (91.9) | 86 (92.5) |  |
| GA/GG | 26 (8.1) | 7 (7.5) |  |
| rs10456242(C/T) |  |  | 0.648 |
| TT | 200 (62.3) | 61 (65.6) |  |
| CT/CC | 121 (37.7) | 32 (34.4) |  |
| rs9358401(G/A) |  |  | 0.467 |
| AA | 94 (29.3) | 23 (24.7) |  |
| GA/GG | 227 (70.7) | 70 (75.3) |  |
| rs7770752(T/G) |  |  | 0.141 |
| GG | 272 (84.7) | 85 (91.4) |  |
| TG/TT | 49 (15.3) | 8 (8.6) |  |
| rs72838053(G/T) |  |  | 0.494 |
| TT | 302 (94.1) | 85 (91.4) |  |
| GT/GG | 19 (5.9) | 8 (8.6) |  |

Abbreviations: APO, adverse pregnancy outcome.

Data was reported n (%).

P for categorical variables derived from Chi-square test or Fisher's exact test.

Appendix Table 2. Unadjusted odds ratios of maternal *CDKAL1* gene variants on APO.

| *CDKAL1* gene variants | OR (95% CI) | *P* |
| --- | --- | --- |
| rs9368268(T/C) |  |  |
| TC/TT vs. CC | 1.25 (0.76,2.04) | 0.376 |
| rs9350258(G/A) |  |  |
| GA/GG vs. AA | 1.10 (0.64,1.87) | 0.735 |
| rs147434309(G/A) |  |  |
| GA/GG vs. AA | 0.77 (0.39,1.51) | 0.440 |
| rs117779648(G/T) |  |  |
| GT/GG vs.TT | 0.72 (0.29,1.80) | 0.484 |
| rs7747752(C/G) |  |  |
| CG/CC vs. GG | 0.76 (0.45,1.26) | 0.287 |
| rs9358361(C/A) |  |  |
| CA/CC vs. AA | 0.83 (0.51,1.35) | 0.460 |
| rs4585542(T/C) |  |  |
| TC/TT vs. CC | 1.03 (0.64,1.67) | 0.908 |
| rs75239109(G/A) |  |  |
| GA/GG vs. AA | 1.50 (0.38,5.90) | 0.566 |
| rs4712573(C/A) |  |  |
| CA/CC vs. AA | 0.90 (0.56,1.44) | 0.650 |
| rs148988294(T/C) |  |  |
| TC/TT vs. CC | 1.25 (0.44,3.55) | 0.681 |
| rs9465946(G/A) |  |  |
| GA/GG vs. AA | 0.85 (0.49,1.47) | 0.561 |
| rs10946425(T/C) |  |  |
| TC/TT vs. CC | 1.03 (0.65,1.65) | 0.888 |
| rs7768086(A/C) |  |  |
| AC/AA vs. CC | 0.95 (0.59,1.52) | 0.817 |
| rs4712585(G/A) |  |  |
| GA/GG vs. AA | 1.21 (0.60,2.44) | 0.593 |
| rs6937439(G/A) |  |  |
| GA/GG vs. AA | 0.85 (0.53,1.37) | 0.499 |
| rs72838040(G/A) |  |  |
| GA/GG vs. AA | 0.92 (0.39,2.20) | 0.858 |
| rs10456242(C/T) |  |  |
| CT/CC vs. TT | 0.87 (0.53,1.41) | 0.563 |
| rs9358401(G/A) |  |  |
| GA/GG vs. AA | 1.26 (0.74,2.14) | 0.391 |
| rs7770752(T/G) |  |  |
| TG/TT vs. GG | 0.52 (0.24,1.15) | 0.105 |
| rs72838053(G/T) |  |  |
| GT/GG vs.TT | 1.50 (0.63,3.54) | 0.359 |

Abbreviations: APO, adverse pregnancy outcome; *CDKAL1*, cyclin-dependent kinase 5 regulatory subunit associated protein 1-like 1; OR, odds ratio; CI, confidence interval.

Appendix Table 3. Clinical characteristics of women with *CDKAL1* genetic marker.

| Characteristic | Non-*CDKAL1* genetic marker | *CDKAL1* genetic marker | *P* |
| --- | --- | --- | --- |
|  | (n=320) | (n=94) |  |
| **Variables during pregnancy** | | | |
| Age, years | 29 [27,31] | 29 [27,31] | 0.821 |
| Pre-pregnancy BMI, kg/m^2^ | 22.5 [20.3,25.0] | 23.2 [20.4,25.4] | 0.457 |
| Systolic BP, mmHg | 105 [100,110] | 105 [100,110] | 0.967 |
| Diastolic BP, mmHg | 70 [60,75] | 70 [60,70] | 0.184 |
| Hypertension | 6 (1.9) | 2 (2.1) | 1.000 |
| Han ethnicity | 311 (97.2) | 91 (96.8) | 1.000 |
| Education >12 years | 172 (53.8) | 55 (58.5) | 0.485 |
| Parity≥1 | 18 (5.6) | 5 (5.3) | 1.000 |
| Family history of diabetes in first degree relatives | 26 (8.1) | 13 (13.8) | 0.143 |
| GDM | 159 (49.7) | 48 (51.1) | 0.907 |
| Gestational age at registration, weeks | 10.0 [9.0,11.0] | 10.0 [9.0,11.0] | 0.805 |
| **Variables during delivery** | | | |
| Infant female gender | 136 (42.5) | 37 (39.4) | 0.672 |
| Birth weight of neonates, g | 3400 [3050,3700] | 3560 [3200,3950] | 0.016 |
| Body height of neonates, cm | 50 [49,51] | 50 [50,52] | 0.001 |
| Delivery week, weeks | 39.0 [38.0,40.0] | 39.0 [38.0,40.0] | 0.890 |
| APO | 60 (18.8) | 33 (35.1) | 0.001 |

Abbreviations: *CDKAL1*, cyclin-dependent kinase 5 regulatory subunit associated protein 1-like 1; BMI, body mass index; BP, blood pressure; GDM, gestational diabetes mellitus; APO: adverse pregnancy outcome.

Data was reported n (%) or medians (IQRs).

P for continuous variables derived from Wilcoxon rank sum test and for categorical variables derived from Chi-square test or Fisher's exact test.
